# Supplementary material for: EnzML: multi-label prediction of enzyme classes using InterPro signatures
Source: BMC Bioinformatics. 2012 Apr 25;13:61. doi: 10.1186/1471-2105-13-61 (PMC3483700; doi:10.1186/1471-2105-13-61)
Supplement: Addtional file 5 — The Java code to format the data files, evaluate and predict. The file enzml_java_code.tar.gz contains the Java code used to format database data to ARFF and XML formats, to execute cross and train-test (jackknife) evaluations and to record evaluation results to database. More information is included in the readme.txt file and the Javadoc files. The code can be used with a MySQL database. To use a different database software, other JDBC drivers might be required. [file 1471-2105-13-61-S5.gz › java_code/utils/doc/uk/ac/ed/inf/utils/database/TableReader.html]

TableReader


---


|  |  |  |
| --- | --- | --- |
| **Overview** \| **Package** \| **Class** \| **Use** \| **Tree** \| **Deprecated** \| **Index** \| **Help** | |  |
| **PREV CLASS**   **NEXT CLASS** | **FRAMES**    **NO FRAMES**     **All Classes** |  |
| SUMMARY: NESTED \| FIELD \| CONSTR \| METHOD | DETAIL: FIELD \| CONSTR \| METHOD |  |


---


## uk.ac.ed.inf.utils.database Class TableReader

```
java.lang.Object
  uk.ac.ed.inf.utils.database.DbManaged
      uk.ac.ed.inf.utils.database.TableManaged
          uk.ac.ed.inf.utils.database.TableReader
```

---

``` public class TableReader extends TableManaged ```

Class

**Version:**
:   10 Jun 2010

**Author:**
:   Luna De Ferrari luna.deferrari-at-ed.ac.uk

---

**Constructor Summary**

`TableReader(TableManager tableManager)`


| **Method Summary** | |
| --- | --- |
| `boolean` | `columnExistsInTable(java.lang.String column)` |
| `TableRow` | `getRowValues(java.sql.ResultSet resultSet, int rowNumber)`             Get the nth row of a `ResultSet` |
| `java.util.Vector<java.lang.String>` | `getTableColumnsNames()`             Gets the list of columns for a database table (from: http://forums.sun.com/thread.jspa?threadID=501222&messageID=2369725) |
| `java.lang.String` | `getTableName()` |
| `TableRow` | `getTableRowWhereEqual(java.lang.String column, java.lang.String value)`             Gets the \*first\* table row where the given column has exactly the given value |
| `TableRow` | `getTableRowWhereLike(java.lang.String column, java.lang.String value)`             Gets the \*first\* table row where the given column has a value similar to the given value |
| `boolean` | `isEmpty(java.sql.ResultSet resultSet)`             Returns true if the resultset is null or empty. |
| `boolean` | `valueExistsInTable(java.lang.String column, java.lang.String value)`             Checks whether a certain row value exists in the given table. |
| `boolean` | `valuesExistsInTable(java.lang.String column1, java.lang.String value1, java.lang.String column2, java.lang.String value2)`             Checks whether a row with the two given values exists in the given table. |

**Methods inherited from class uk.ac.ed.inf.utils.database.TableManaged**

`getTableManager`

**Methods inherited from class uk.ac.ed.inf.utils.database.DbManaged**

`getDbConnection, getDbManager`

**Methods inherited from class java.lang.Object**

`equals, getClass, hashCode, notify, notifyAll, toString, wait, wait, wait`

**Constructor Detail**

### TableReader

```
public TableReader(TableManager tableManager)
```


**Method Detail**

### columnExistsInTable

```
public boolean columnExistsInTable(java.lang.String column)
```

---


### getRowValues

```
public TableRow getRowValues(java.sql.ResultSet resultSet,
                             int rowNumber)
```

:   Get the nth row of a `ResultSet`

    :   **Parameters:**: `resultSet` -: `rowNumber` - (starting at 0) **Returns:**: the row

---


### getTableColumnsNames

```
public java.util.Vector<java.lang.String> getTableColumnsNames()
```

:   Gets the list of columns for a database table (from:
    http://forums.sun.com/thread.jspa?threadID=501222&messageID=2369725)

    :   **Returns:**: a list of table columns

---


### getTableName

```
public java.lang.String getTableName()
```

---


### getTableRowWhereEqual

```
public TableRow getTableRowWhereEqual(java.lang.String column,
                                      java.lang.String value)
```

:   Gets the \*first\* table row where the given column has exactly the given
    value

    :   **Parameters:**: `column` - a table column name: `value` - a value to be found in that column **Returns:**: the \*first\* table row where the given column has exactly the given value

---


### getTableRowWhereLike

```
public TableRow getTableRowWhereLike(java.lang.String column,
                                     java.lang.String value)
```

:   Gets the \*first\* table row where the given column has a value similar to
    the given value

    :   **Parameters:**: `column` - a table column name: `value` - a value to be found in that column **Returns:**: the \*first\* table row where the given column has a value similar to the given value

---


### isEmpty

```
public boolean isEmpty(java.sql.ResultSet resultSet)
```

:   Returns true if the resultset is null or empty. Emptyness checked using
    the method: if resultSet.isBeforeFirst() is false, then the resultset
    must be empty http://forums.sun.com/thread.jspa?threadID=481745

    :   **Parameters:**: `resultSet` - **Returns:**: true if the resultset is null or empty

---


### valueExistsInTable

```
public boolean valueExistsInTable(java.lang.String column,
                                  java.lang.String value)
```

:   Checks whether a certain row value exists in the given table.

    :   **Parameters:**: `value` - the row value: `column` - the table column **Returns:**: true if a row exists with that column value

---


### valuesExistsInTable

```
public boolean valuesExistsInTable(java.lang.String column1,
                                   java.lang.String value1,
                                   java.lang.String column2,
                                   java.lang.String value2)
```

:   Checks whether a row with the two given values exists in the given table.

    :   **Parameters:**: `value` - the row value: `column` - the table column **Returns:**: true if a row exists with that column value


---


|  |  |  |
| --- | --- | --- |
| **Overview** \| **Package** \| **Class** \| **Use** \| **Tree** \| **Deprecated** \| **Index** \| **Help** | |  |
| **PREV CLASS**   **NEXT CLASS** | **FRAMES**    **NO FRAMES**     **All Classes** |  |
| SUMMARY: NESTED \| FIELD \| CONSTR \| METHOD | DETAIL: FIELD \| CONSTR \| METHOD |  |


---
